# Efficacy and safety of elobixibat in combination with or switched from conventional treatments of chronic constipation: A retrospective observational study

**DOI:** 10.1002/jgh3.70019

**Published:** 2024-08-26

**Authors:** Takaaki Eguchi, Osamu Inatomi, Shuhei Shintani, Kenji Momose, Tomoya Sako, Megumi Takagi, Daiki Fumihara, Kazuki Inoue, Norio Katayama, Toshiyuki Morisawa, Takumi Ota, Yoshihisa Tsuji

**Affiliations:** ^1^ Department of Gastroenterology and Hepatology Osaka Saiseikai Nakatsu Hospital Osaka Japan; ^2^ Department of General Medicine Shiga University of Medical Science Hospital Shiga Japan; ^3^ Division of Gastroenterology, Department of Medicine Shiga University of Medical Science Shiga Japan; ^4^ Medical Affairs Department Mochida Pharmaceutical Co., Ltd. Tokyo Japan

**Keywords:** Bristol stool form scale, chronic constipation, elobixibat, laxative refractory, spontaneous bowel movement

## Abstract

**Background and Aim:**

Elobixibat is a triple mode of action laxative that increases water secretion into the colon, promotes colonic motility, and reestablishes the defecation desire. This study aims to evaluate the effectivity and safety of elobixibat in chronic constipation (CC) patients refractory to conventional laxatives.

**Methods:**

A single‐center retrospective observational study was conducted in refractory CC patients diagnosed according to the Rome IV criteria and received elobixibat between April 2018 and June 2022 at Osaka Saiseikai Nakatsu Hospital. Data were collected for spontaneous bowel movement (SBM), Bristol stool form scale (BSFS) scores, abdominal symptoms, and adverse events.

**Results:**

Eligible 311 patients were selected for the analysis. Two‐week Elobixibat treatment significantly increased SBM (times/week) from 2.9 ± 1.9 to 4.3 ± 1.9 (*P* < 0.0001). The BSFS score improved significantly from 3.2 ± 1.7 to 4.4 ± 1.4 (*P* < 0.0001). The percentages of patients with hard stool were decrease and that with normal stools were increase. Improvements in abdominal symptoms (sensation of incomplete bowel evacuation, straining, abdominal pain and distention, and difficulty defecating) were also significant (*P* < 0.05). These constipation symptoms were improved irrespective of patient characteristics or previous laxatives. The 43.9% of previous laxatives were discontinued at the start of or after starting elobixibat treatment. A few adverse events were observed, elobixibat was well tolerated.

**Conclusion:**

Elobixibat was effective in patients who were refractory to other laxatives, irrespective of previous therapy or patient characteristics. Elobixibat may contribute to resolving polypharmacy with single mode of action laxatives.

## Graphical abstract

A retrospective observational study was conducted to evaluate the effectivity and safety of elobixibat in patients with chronic constipation who were refractory to conventional laxatives. 2‐week Elobixibat treatment significantly increased spontaneous bowel movement. The percentages of patients with hard stool were decrease, and that with normal stools were increase. Improvements in abdominal symptoms (sensation of incomplete bowel evacuation, straining, abdominal pain and distention, and difficulty defecating) were significant. These constipation symptoms were improved irrespective of patient characteristics or previous laxatives.

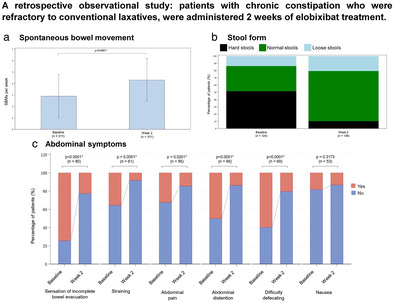

## Introduction

Chronic constipation (CC) is a common gastrointestinal symptom, resulting in reduced quality of life^1^ and labor productivity^2^ and a poorer prognosis.^3^ Approximately 16%^4^ of the global population is estimated to experience CC. CC is more common in women and older individuals,^5^ accompanying various type of disease.^6^, ^7^, ^8^, ^9^, ^10^ Constipation has multiple causes, including diminished peristalsis, decreased water intake, and impaired rectal sensation.^11^ Symptoms of CC are diverse and can include infrequent bowel movement, hard stools, straining, abdominal pain and distention, difficulty defecating, and loss of the desire to defecate.^12^, ^13^

Laxatives are broadly divided by their mechanism of pharmacologic action into “agents that add water and soften stools” (osmotic laxatives and intestinal secretagogues) and “agents that promote motility of the large intestine” (stimulant laxatives and gastrointestinal prokinetic agents),^14^ both of which are applicable to elobixibat.^15^ In Japan, magnesium oxide and stimulant laxatives have been used to treat constipation. However, these laxatives often fail to normalize stool form,^16^ and moreover, magnesium oxide and stimulant laxatives respectively pose the risk of hypermagnesemia^17^ and acquired resistance after prolonged use.^18^ Some novel laxatives such as intestinal secretagogues (lubiprostone and linaclotide) and a bile acid transporter inhibitor (elobixibat) have made multiple treatment options available for CC; however, approximately 50% of patients with CC remain dissatisfied with their current laxatives.^19^

Elobixibat inhibits ileal bile acid transporters (also called apical sodium‐dependent bile acid transporters), which contribute to bile acid reabsorption.^20^ Elobixibat prevents bile acid reabsorption, thereby increasing the amount of bile acid flowing into the large intestinal lumen and upregulating bile acid synthesis by the liver.^21^ Bile acids that have increased in amount in the large intestine activate transmembrane G protein‐coupled receptor five and thereby increase water and electrolyte secretion into the colon and induce high‐amplitude propagated contractions, resulting in increased frequency of colonic motility.^20^, ^21^ Furthermore, elobixibat improves the defecation desire in patients with CC,^22^ resulting from the decrease in the rectal sensory threshold due to the increase in bile acids.^21^, ^23^

This triple mode of action laxative is presumed to be more effective in patients with CC than single mode of action laxatives such as magnesium oxide, stimulant laxatives, intestinal secretagogues, and prokinetics.^24^, ^25^, ^26^, ^27^, ^28^, ^29^ However, it is unknown whether elobixibat can be used effectively and safely in patients who were refractory to these, single mode of action laxatives. Demonstration of efficacy of elobixibat in patients who are refractory to single mode of action laxatives may establish a new treatment option. Thus, this study evaluated the efficacy and safety of elobixibat in patients with CC who were refractory to conventional laxatives.

## Methods

### Study design

The Ethics Committee of Osaka Saiseikai Nakatsu Hospital approved this study (approval No. 2022‐33). The study was conducted in accordance with the Helsinki Declaration. The study was retrospective, and it was therefore not possible to obtain patients' written informed consent. However, study information was posted on the hospital website to ensure an opportunity for patients to opt out of the use of their information, for example, medical records used for the study. This consent procedure was approved by the Ethics Committee of Osaka Saiseikai Nakatsu Hospital. The study was registered on the University Hospital Medical Information Network (UMIN) (UMIN000049639).

A retrospective analysis was performed based on the dataset of medical records without personal information, in patients who received elobixibat between 19 April 2018 and 30 June 2022 at the hospital and met the following eligibility criteria: (i) 18 years of age or older; (ii) patients who were determined to be refractory to other laxatives (defined as being judged by a doctor to be unsuitable in terms of efficacy and safety for treatment with various laxatives [magnesium oxide, stimulant laxatives, intestinal secretagogues, etc.] or Chinese traditional medicines for constipation) by meeting, despite treatment, at least two of the following Rome IV criteria,^30^ that is, decreased frequency of bowel movements (<3 times/week), sensation of incomplete evacuation, hard stools, or a history of difficult evacuation on at least one of four occasions; (iii) patients with CC diagnosed with Rome IV criteria^30^ who were prescribed other laxatives for at least 1 week, followed by elobixibat treatment with documentation of the course of treatment for a duration of at least 2 weeks, and the frequency of bowel movements before and after treatment; (iv) patients not hypersensitive to elobixibat; (v) patients who did not have intestinal obstruction; (vi) patients whose prescriptions of elobixibat were not outside the approved indications or dosage and administration during the observation period (defined as the observations from baseline to Week 2); and (vi) patients who did not participate in any other clinical trials including interventional studies during the observation period. All patient data including age, sex, comorbidities, prior laxatives, and chief complaint were collected from the electronic database. The patient‐reported Bristol stool form scale (BSFS) for the past one or two weeks that recorded in the medical record through direct interview with patients was collected. If a patient was not able to report their BSFS, the investigators collected the BSFS that the medical provider verified. All the laxatives were prescribed in accordance with the approved dosage and administration. Patients were received 10 mg (2 tablets of 5 mg each) of elobixibat orally once daily before meals. Elobixibat could be dose‐adjusted between 5 and 15 mg according to the degree of symptoms. The decision to switch from prior laxatives to elobixibat or to add elobixibat to prior laxatives was based on criteria such as if symptoms did not improve, worsened, or if adverse events were observed with prior laxatives. Criteria for discontinuing concomitant laxatives during elobixibat treatment included excessive stools, frequent stools, or presumed adverse events related to the concomitant laxatives. The decision to discontinue the prior laxatives was left to the patients and doctors' discretion.

### Outcomes

The primary outcome was the change in spontaneous bowel movement (SBM; defined as the weekly frequency of bowel movements without enemas or stool extraction) from baseline to Week 2. In addition, the percentage of responders, as defined by SBM ≥3 times/week at Week 2 with a change in SBM of ≥1 time/week (Week 2 − baseline), was calculated.

Secondary endpoints included a comparison of BSFS scores^31^ for stool form, sensation of incomplete bowel evacuation, straining, abdominal pain and distention, difficulty defecating, and the presence or absence of nausea at baseline and Week 2. The incidences of adverse events and adverse reactions and the elobixibat treatment discontinuation rate were calculated. In addition, the change from baseline in SBM and BSFS and the presence or absence of the sensation of incomplete bowel evacuation before and after administration were analyzed for the prespecified subgroups based on age, self‐reported sex, comorbidities (yes/no), laxatives initiated before the prescription of elobixibat (yes/no), BSFS before elobixibat, use of magnesium before the prescription of elobixibat (yes/no) and baseline SBM, and use of intestinal secretagogues before the prescription of elobixibat (yes/no) and baseline SBM. Furthermore, the factors that affect the responder status (yes/no) and adverse reactions (yes/no) were explored.

### Statistical analysis

Analysis data sets were prepared, and statistical analyses were performed using SAS version 9.4 software (SAS Institute Inc., Carey, NC, USA). All adverse events that occurred were coded using the Medical Dictionary for Regulatory Activities/Japanese (MedDRA/J) version 25.1. Numeric variables were expressed as mean ± standard deviation. The changes in SBM and in stool consistency were compared using a paired *t*‐test and the Wilcoxon signed rank test, respectively, and the changes in the sensation of incomplete bowel evacuation, straining, abdominal pain and distention, difficulty defecating, and nausea were compared using McNemar's test. The odds ratio and its 95% confidence interval (CIs) were estimated for each factor by fitting a logistic‐regression model with the responder status (yes/no) and adverse reactions (yes/no) as outcome variables and age (≥65 or <65 years), sex, comorbidities (yes/no), use of each type of laxative before the prescription of elobixibat (yes/no), and the number of types of laxatives used before the prescription of elobixibat (one, two, or three types or more) as explanatory variables. Statistical significance was defined as *P* < 0.05.

Statistical analyses for the study were performed by an independent contractor (SRD Co., Ltd.; Tokyo, Japan).

## Results

### Patients

**Figure 1:**
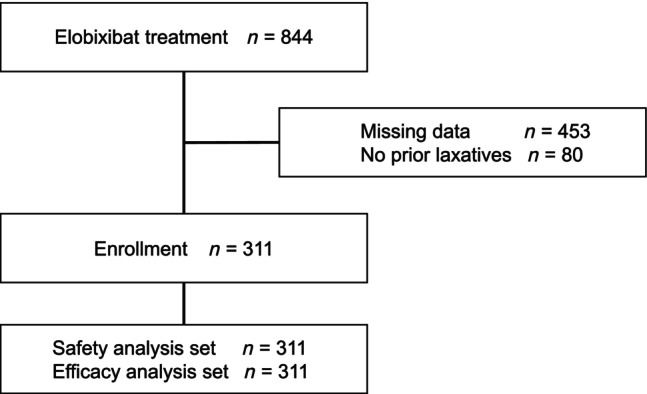
Flow chart of patient enrolment.

Of 844 patients who received elobixibat at the hospital between 19 April 2018, and 30 June 2022, eligible 311 patients were selected for the analysis, excluding 453 patients with missing data for the frequency of bowel movements before and after elobixibat and 80 patients who had not previously used prescribed laxatives (Fig. 1). All 311 patients were included in the efficacy and safety analyses. Table 1 shows the patient background. The starting dose of elobixibat was 5 mg/day in 89 patients (28.6%), 10 mg/day in 212 patients (68.2%), and 15 mg/day in 10 patients (3.2%).

**Table 1:** Patient characteristics

	*n* = 311
Age (years) (range)	74.5 ± 13.3 (21–101)
Range of years old [*n* (%)]	
≦34	6 (1.9)
35–49	9 (2.9)
50–64	38 (12.2)
65–74	70 (22.5)
≧75	188 (60.5)
Men [*n* (%)]	159 (51.1)
Duration of chronic constipation (years)	1.4 ± 2.5
Number of types of prior laxatives for chronic constipation	2.4 ± 1.3
Prior laxatives for chronic constipation [*n* (%)]	
Stimulant laxatives	166 (53.4)
Osmotic laxatives	161 (51.8)
Intestinal secretagogues	116 (37.3)
Suppositories/enemas	65 (20.9)
Chinese traditional medicines	46 (14.8)
Gastrointestinal prokinetic agents	32 (10.3)
Other	55 (17.7)
Comorbidities [*n* (%)]	
Yes	298 (95.8)
Cardiovascular diseases	155 (49.8)
Cancer	121 (38.9)
Diabetes mellitus	81 (26.0)
Chronic kidney failure	64 (20.6)
Postabdominal surgery	51 (16.4)
Cerebral infarction/hemorrhage	43 (13.8)
Orthopedic diseases	42 (13.5)
Dementia	35 (11.3)
Respiratory diseases	29 (9.3)
Use of opioids	28 (9.0)
Dialysis	23 (7.4)
Neurologic diseases	23 (7.4)
Infections and infestations	20 (6.4)
Chronic hepatitis	19 (6.1)
Psychiatric diseases	18 (5.8)
Gastrointestinal disorders	17 (5.5)
Renal and urinary disorders	14 (4.5)
Endocrine diseases	11 (3.5)
Collagen disorders	5 (1.6)
Rectal ulcer	4 (1.3)

Data in the table represent mean ± standard deviation unless otherwise specified.

### Primary outcome

**Figure 2:**
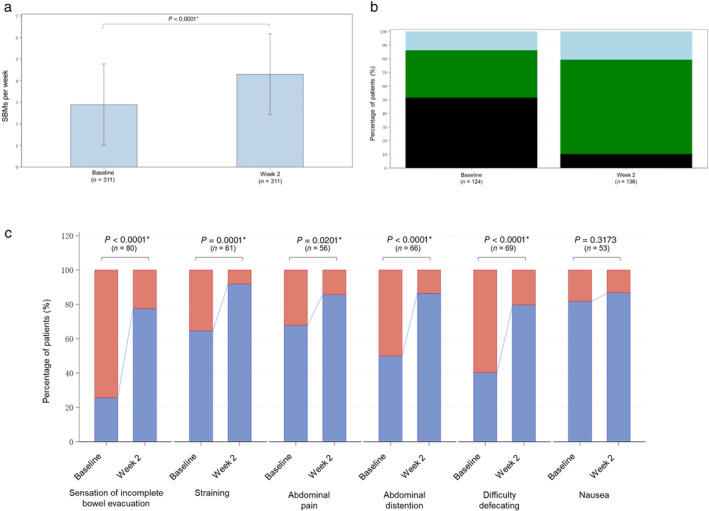
(a) Change in weekly spontaneous bowel movement (SBM) frequency from baseline to Week 2 of elobixibat treatment. Each bar and error bar indicate mean and standard deviation. *P < 0.05 *versus* baseline. (b) Change in the percentage of stool form based on the Bristol stool form scale (BSFS) from baseline to Week 2 of elobixibat treatment. BSFS scores 1–2 indicate hard stools (■), scores 3–5 indicate normal stools (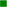), and scores 6–7 indicate loose stools (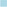). (c) Change in the percentages of patients with (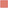 Yes) and without (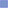 No) each abdominal symptom from baseline to Week 2 of elobixibat treatment. *P < 0.05 *versus* baseline.

Following 2‐week elobixibat treatment, mean SBM (times/week) significantly increased from 2.9 ± 1.9 times/week at baseline to 4.3 ± 1.9 times/week at Week 2 (*P* < 0.0001, paired *t*‐test, Fig. 2a). The percentage of responders in terms of SBM was 60.1% (187/311 patients).

### Secondary outcomes

#### Efficacy

Following 2‐week elobixibat treatment, the mean BSFS score significantly increased from 3.2 ± 1.7 at baseline to 4.4 ± 1.4 at Week 2 (*P* < 0.0001, Wilcoxon signed rank test). The percentage of hard stools (BSFS: 1–2) decreased from 51.6% (64/124 patients) at baseline to 10.3% (14/136 patients) at Week 2, and the percentage of normal stools (BSFS: 3–5) increased from 34.7% (43/124 patients) at baseline to 69.1% (94/136 patients) at Week 2. The percentage of loose stools (BSFS: 6–7) was 13.7% (17/124 patients) at baseline and 20.6% (28/136 patients) at Week 2 (Fig. 2b).

Elobixibat treatment significantly improved in abdominal symptoms; the sensation of incomplete bowel evacuation, straining, abdominal pain and distention, and difficulty defecating (*P* < 0.05, McNemar's test). Although no statistically significant change in nausea was observed (*P* = 0.3173, McNemar's test), the percentage of patients with nausea decreased from 17.0% (9/53 patients) at baseline to 13.2% (7/53 patients) at Week 2 (Fig. 2c and Table S1, Supporting information).

During the observation period, 43.9 ± 40.7% of previously used laxatives were discontinued. The most common laxatives were enemas and suppositories (78.5%, 51/65 patients), followed by stimulant laxatives (62.0%, 103/166 patients) (Table 2). Forty‐two patients discontinued all laxatives previously used before receiving elobixibat (Table 4).

**Table 2:** Percentage of patients who discontinued other laxatives during elobixibat treatment

	Initiated before elobixibat treatment	Discontinued during the observation period^†^	Switched to elobixibat^⁑^	Discontinued during elobixibat treatment^§^
Osmotic laxatives	161	50 (31.1)	37 (23.0)	14 (8.7)
Magnesium	150	48 (32.0)	34 (22.7)	14 (9.3)
Stimulant laxatives	166	103 (62.0)	66 (39.8)	38 (22.9)
Intestinal secretagogues	116	50 (43.1)	40 (34.5)	10 (8.6)
Gastrointestinal prokinetic agents	32	2 (6.3)	1 (3.1)	1 (3.1)
Enemas and suppositories	65	51 (78.5)	33 (50.8)	21 (32.3)
Chinese traditional medicines	46	16 (34.8)	11 (23.9)	5 (10.9)

^†^ Laxatives initiated before elobixibat treatment that were discontinued at the start of elobixibat treatment or by the end of the observation period.

^⁑^ Laxatives initiated before elobixibat treatment that were discontinued on starting elobixibat.

^§^ Laxatives initiated before elobixibat treatment that were discontinued by the end of the observation period.

(%) represents the ratio to the number of persons who had initiated each laxative before elobixibat treatment.

### Discontinuation rate and safety

The incidence of adverse events was 6.1% (19/311 patients, 20 events), comprising 3.9% (12/311) diarrhea, 1.6% (5/311) abdominal pain, 0.6% (2/311) abdominal distention, and 0.3% (1/311) nausea. These events were at least possibly related to elobixibat, that is, adverse reactions, in 5.8% (18/311) of the patients, except for abdominal pain in one patient. These were all mild‐to‐moderate in severity, with no serious adverse events. All adverse reactions recovered with elobixibat dose reduction or treatment discontinuation. Adverse events led to treatment discontinuation in 12 patients. Twenty‐two patients discontinued treatment owing to improvement in constipation.

### Factors that affect responder status and adverse reactions

There was no significant correlation between responder status or adverse reactions and the following factors: age, sex, comorbidities (yes/no), use of each type of laxative before the prescription of elobixibat (yes/no), and the number of types of such laxatives (Table 3).

**Table 3:** Factors related to responder status and the occurrence of adverse reactions

Explanatory variable	Odds ratio for responder status (yes/no)^†^		Odds ratio for the occurrence of adverse reactions (yes/no)	
	Odds ratio	95% confidence interval	Odds ratio	95% confidence interval
Age (≥65/<65 years)	0.651	[0.324–1.273]	0.581	[0.179–2.174]
Sex (male/female)	0.825	[0.505–1.347]	0.705	[0.239–1.993]
Comorbidities (yes/no)	0.349	[0.051–1.448]	0.325	[0.061–2.615]
Pretreatment (yes/no)				
Osmotic laxatives	1.239	[0.294–6.481]	4.630	[0.483–34.403]
Magnesium	0.409	[0.076–1.768]	0.663	[0.089–6.679]
Stimulant laxatives	0.851	[0.439–1.642]	1.557	[0.416–6.080]
Intestinal secretagogues	0.767	[0.402–1.466]	1.508	[0.402–5.485]
Chinese traditional medicines	0.697	[0.321–1.521]	2.151	[0.397–9.335]
Enemas or suppositories	1.776	[0.905–3.577]	1.679	[0.397–6.364]
Gastrointestinal prokinetic agents	1.009	[0.419–2.497]	1.476	[0.183–7.948]
Number of types of pretreatment laxatives				
2 types/1 type	1.060	[0.461–2.445]	0.711	[0.125–3.804]
≥ 3 types/1 type	2.582	[0.607–11.278]	0.226	[0.009–4.224]

^†^ “Responder” was defined by spontaneous bowel movement (SBM) ≥3 times/week at Week 2 with a change in SBM (Week 2 − baseline) of ≥1 time/week, and other cases were regarded as “nonresponders.”

Each explanatory variable (A/B) represents the odds ratio for level A to level B.

### Subgroup analyses

Results are presented in Table 4 for the analysis of SBM, BSFS, and the sensation of incomplete bowel evacuation (yes/no) for the prespecified subgroups based on patient characteristics or prior treatment (Table S2).

**Table 4:** Subgroup analysis of spontaneous bowel movement (SBM), Bristol stool form scale (BSFS) scores, and the sensation of incomplete bowel evacuation.

Patient characteristics	SBM, mean ± SD, (*n*)		BSFS, mean ± SD, (*n*)		Sensation of incomplete bowel evacuation, *n* (%)	
	Baseline	Week 2	Baseline	Week 2	Baseline	Week 2
Age						
≥65 years	2.9 ± 1.8 (258)	4.3 ± 1.9* (258)	3.1 ± 1.8 (103)	4.4 ± 1.3* (110)	51 (81.0)	15 (23.8)*
<65 years	3.0 ± 2.2 (53)	4.5 ± 1.8* (53)	3.4 ± 1.6 (21)	4.5 ± 1.6 (26)	9 (52.9)	3 (17.6)*
Sex						
Male	3.0 ± 1.9 (159)	4.3 ± 1.9* (159)	3.0 ± 1.7 (53)	4.4 ± 1.4* (56)	25 (78.1)	6 (18.8)*
Female	2.8 ± 1.8 (152)	4.4 ± 1.8* (152)	3.3 ± 1.7 (71)	4.4 ± 1.3* (80)	35 (72.9)	12 (25.0)*
Baseline SBM (times/week)						
≥3	4.7 ± 1.5 (133)	5.1 ± 1.8* (133)	3.3 ± 1.9 (60)	4.5 ± 1.3* (68)	29 (76.3)	9 (23.7)*
<3	1.6 ± 0.5 (178)	3.7 ± 1.7* (178)	3.1 ± 1.5 (64)	4.2 ± 1.4* (68)	31 (73.8)	9 (21.4)*
Baseline BSFS						
1–2	3.1 ± 1.9 (64)	4.6 ± 1.9* (64)	1.8 ± 0.4 (64)	3.8 ± 1.3* (59)	24 (80.0)	7 (23.3)*
3–5	3.0 ± 2.0 (43)	4.5 ± 1.8* (43)	4.0 ± 0.8 (43)	4.4 ± 1.1* (38)	15 (65.2)	4 (17.4)*
6–7	4.0 ± 2.1 (17)	4.6 ± 1.9 (17)	6.3 ± 0.5 (17)	5.0 ± 1.0* (17)	3 (42.9)	1 (14.3)
Comorbidities						
Cardiovascular diseases	2.8 ± 1.8 (155)	4.3 ± 1.9* (155)	2.9 ± 1.7 (56)	4.3 ± 1.3* (61)	30 (81.1)	12 (32.4)*
Cancer	3.1 ± 1.9 (121)	4.4 ± 1.8* (121)	3.2 ± 1.7 (45)	4.3 ± 1.3* (42)	19 (76.0)	2 (8.0)*
Diabetes mellitus	3.0 ± 1.8 (81)	4.1 ± 1.9* (81)	3.1 ± 1.8 (30)	4.5 ± 1.3* (31)	17 (81.0)	5 (23.8)*
Postabdominal surgery	3.2 ± 2.2 (51)	4.2 ± 2.1* (51)	3.7 ± 1.6 (19)	4.4 ± 1.3 (22)	11 (78.6)	4 (28.6)*
Cerebral infarction/hemorrhage	2.4 ± 1.9 (43)	3.8 ± 1.9* (43)	3.3 ± 2.0 (13)	4.9 ± 1.1 (13)	5 (71.4)	4 (57.1)
Dementia	2.4 ± 1.8 (35)	3.5 ± 1.8* (35)	3.6 ± 1.6 (14)	4.5 ± 1.0 (15)	7 (100.0)	3 (42.9)*
Use of opioids	3.1 ± 1.7 (28)	4.7 ± 1.8* (28)	2.8 ± 1.8 (5)	4.2 ± 1.3 (5)	2 (66.7)	0 (0.0)
Number of types of prior laxatives						
1 type	2.8 ± 1.8 (85)	4.3 ± 2.0* (85)	3.3 ± 1.7 (34)	4.4 ± 1.4* (39)	15 (78.9)	5 (26.3)*
2 types	2.9 ± 1.9 (82)	4.2 ± 1.8* (82)	3.0 ± 1.6 (34)	4.3 ± 1.3* (37)	15 (65.2)	4 (17.4)*
≥3 types	2.9 ± 1.9 (132)	4.3 ± 1.9* (132)	3.3 ± 1.8 (51)	4.4 ± 1.4* (53)	25 (78.1)	7 (21.9)*
Prior laxatives						
Osmotic laxatives	3.0 ± 1.9 (161)	4.4 ± 1.8* (161)	3.3 ± 1.8 (68)	4.4 ± 1.4* (72)	35 (74.5)	9 (19.1)*
Magnesium	3.1 ± 1.9 (150)	4.4 ± 1.8* (150)	3.2 ± 1.8 (62)	4.4 ± 1.3* (67)	30 (75.0)	8 (20.0)*
Stimulant laxatives	2.8 ± 1.8 (166)	4.1 ± 1.9* (166)	3.4 ± 1.7 (66)	4.5 ± 1.3* (70)	32 (78.0)	10 (24.4)*
Intestinal secretagogues	2.9 ± 1.9 (116)	4.3 ± 1.8* (116)	3.1 ± 1.7 (50)	4.2 ± 1.6* (52)	18 (66.7)	7 (25.9)*
Chinese traditional medicines	2.5 ± 1.5 (46)	3.8 ± 1.9* (46)	3.6 ± 1.9 (15)	4.5 ± 1.3 (17)	6 (85.7)	0 (0.0)*
Enemas/suppositories	2.8 ± 1.8 (65)	4.4 ± 1.8* (65)	3.0 ± 1.9 (28)	4.3 ± 1.5* (30)	18 (100.0)	4 (22.2)*
Gastrointestinal prokinetic agents	2.5 ± 1.7 (32)	4.4 ± 1.9* (32)	3.4 ± 1.6 (10)	4.5 ± 0.7* (11)	2 (33.3)	0 (0.0)
Intestinal secretagogues and osmotic laxatives	3.1 ± 1.9 (57)	4.4 ± 1.7* (57)	3.4 ± 1.8 (23)	4.1 ± 1.8 (23)	10 (71.4)	4 (28.6)*
Intestinal secretagogues and magnesium	3.1 ± 1.9 (53)	4.3 ± 1.7* (53)	3.4 ± 1.9 (21)	4.2 ± 1.7 (22)	9 (69.2)	3 (23.1)*
Intestinal secretagogues and stimulant laxatives	2.9 ± 2.0 (62)	4.2 ± 1.7* (62)	3.5 ± 1.8 (29)	4.5 ± 1.4* (31)	11 (68.8)	4 (25.0)*
Prior laxatives and baseline SBM (times/week)						
Magnesium and baseline SBM ≥3	4.7 ± 1.5 (72)	5.2 ± 1.6* (72)	3.5 ± 2.0 (30)	4.6 ± 1.3* (36)	16 (80.0)	4 (20.0)*
Magnesium and baseline SBM< 3	1.6 ± 0.5 (78)	3.6 ± 1.7* (78)	3.0 ± 1.5 (32)	4.2 ± 1.3* (31)	14 (70.0)	4 (20.0)*
Intestinal secretagogues and baseline SBM ≥3	4.7 ± 1.5 (51)	4.9 ± 1.8 (51)	3.5 ± 1.9 (27)	4.3 ± 1.6 (29)	10 (71.4)	3 (21.4)*
Intestinal secretagogues and baseline SBM<3	1.5 ± 0.5 (65)	3.7 ± 1.7* (65)	2.7 ± 1.4 (23)	4.0 ± 1.7* (23)	8 (61.5)	4 (30.8)*
Switching from prior laxatives						
Switching from all of prior laxatives to elobixibat	2.8 ± 2.0 (42)	4.6 ± 1.7* (42)	3.0 ± 1.5 (24)	4.3 ± 1.2* (27)	11 (68.8)	3 (18.8)*

* *P* < 0.05.

The changes from baseline in the frequency of SBMs and BSFS scores were measured using the Paired *t*‐test and the Wilcoxon signed rank test, respectively. Marginal probability of the sensation of incomplete bowel evacuation before and after treatment was calculated using the McNemar's test.

SD, standard deviation.

SBM, BSFS, and the sensation of incomplete bowel evacuation were significantly improved in all subgroups based on self‐reported sex and baseline SBM. In patients with loose stools at baseline, BSFS scores significantly reduced, with the mean score within the range of normal stools. In all subgroups based on comorbidity and age, all or any of SBM, BSFS, and the sensation of incomplete bowel evacuation were significantly improved.

Significant improvements in all or any of SBM, BSFS, and the sensation of incomplete bowel evacuation were observed regardless of the number of prior laxatives and types of prior laxatives. In patients who no longer required other laxatives following the prescription of elobixibat, significant improvements were observed in SBM, BSFS, and the sensation of incomplete bowel evacuation.

In patients who used multiple laxatives before receiving elobixibat, significant improvements were observed in all or any of SBM, BSFS, and the sensation of incomplete bowel evacuation.

In patients who used intestinal secretagogues or magnesium before receiving elobixibat but had baseline SBM of <3 times/week, significant improvements were observed in SBM, BSFS, and the sensation of incomplete bowel evacuation. In patients who used intestinal secretagogues or magnesium before receiving elobixibat and had baseline SBM of ≥3 times/week, sensation of incomplete bowel evacuation was improved significantly.

In patients who used laxatives before receiving elobixibat and discontinued all laxatives after receiving elobixibat, significant improvements were observed in SBM, BSFS, and the sensation of incomplete bowel evacuation.

## Discussion

Despite this being a single‐center retrospective study, as many as 311 patients were included in an analysis that evaluated the efficacy and safety of elobixibat in patients with CC refractory to conventional laxatives. A significant improvement in SBM was observed 2 weeks after elobixibat treatment. In the previous retrospective observational study conducted at a single center in Japan, which used similar design to our study in terms of doses, administration methods, and observation periods, the patients' mean age was 77.7 years. This previous study observed improvements in stool frequency and BSFS after 2 weeks of elobixibat administration and noted that 36.9% of patients switched to elobixibat.^32^ In addition, the efficacy was observed regardless of concomitant medications in the previous study. The efficacy of elobixibat in the elderly patients of our study were consistent with these results. According to an interim analysis of post‐marketing surveillance data,^33^ this study showed similar efficacy for SBM, although 33.4% of the survey participants had received no prior treatment, unlike the present study. This study demonstrated that elobixibat exerts its efficacy from an early phase of treatment. The percentage of responders in respect of SBM was more than half. A significant improvement was observed in stool consistency 2 weeks after treatment. Significant improvements were also observed in constipation symptoms other than nausea. Taken together, the study demonstrated the efficacy of elobixibat for various constipation symptoms that had responded poorly to prior laxatives. Although this study could not demonstrate a significant improvement in nausea owing to the limited number of patients with nausea at baseline, the percentage of patients with nausea decreased with treatment. Nausea newly occurred in one of the 53 patients (1.9%) after elobixibat treatment, suggesting a low potential of elobixibat to induce nausea.

The study also investigated patient characteristics related to responder status in terms of SBM. The study revealed no factors significantly related to responder status, suggesting that elobixibat is effective in CC patients with a wide range of characteristics. A subgroup analysis according to patient characteristics showed improvements in constipation symptoms as an increase in SBM, an improvement in stool consistency, or a decrease in the sensation of incomplete bowel evacuation across all subgroups. In general, older individuals and women have diminished gastrointestinal motility^34^, ^35^ and are more susceptible to constipation. In this study, an increase in SBM was observed in patients ≥65 years of age and women, which may result from elobixibat promoting gastrointestinal motility that had been diminished in such individuals. A significant improvement was observed in BSFS in patients with hard stools (BSFS: 1–2) at baseline, with an increase in the mean BSFS score into the range of normal stools 2 weeks after treatment. This is partly attributable to water secretion by elobixibat into the large intestinal lumen. In patients with loose stools (BSFS: 6–7) at baseline, a significant improvement was observed in BSFS, with a decrease in the mean BSFS score into the range of normal stools 2 weeks after treatment. Prior laxatives were discontinued during the observation period in 13 of the 17 patients with loose stools. After switching to elobixibat, patients with CC who had loose stools poorly controlled by other laxatives achieved moderate stool consistency. In this study, the dose of elobixibat was able to decrease based on the symptom. Therefore, dose decrease might affect the normalization of high BSFS scores. On the other hand, it is believed that elobixibat, with a milder pharmacologic action than the prior treatments, allowed bowel movements to more closely approximate physiological bowel movements.

Constipation has been reported to be associated with Parkinson's disease,^6^ dementia,^7^ depression,^8^ diabetes mellitus,^10^ chronic kidney disease^9^ and use of opioids.^36^ This study demonstrated improvements in constipation symptoms with all the comorbidities present in the study, shown by improvement in SBM, BSFS, or the sensation of incomplete bowel evacuation. Elobixibat has been reported to be effective in improving constipation in patients with concurrent diabetes mellitus and cancer,^37^, ^38^ and the results of the present study were consistent with these previous reports. In the patients with diabetes mellitus and those on opioids, elobixibat may have increased previously diminished motility in the large intestine,^10^, ^36^ leading to the improvement in constipation symptoms. In patients with dementia, who may develop aggressive behavior due to difficulty expressing in words their pain and discomfort with constipation,^7^ treatment of constipation is considered important; however, there are few reports that demonstrate an improvement due to laxatives. This study demonstrated the efficacy of elobixibat in improving constipation in patients with dementia, indicating the utility of elobixibat in patients with dementia and CC.

In this study, elobixibat improved constipation symptoms even in patients who were refractory to conventional laxatives. Magnesium oxide, a common laxative in Japan, poses the risk of hypermagnesemia especially in older individuals with reduced renal function.^17^ Given that osmotic laxatives such as magnesium oxide promote water secretion, while elobixibat increases motility in the large intestine and reestablishes a desire to defecate, it is considered reasonable to use elobixibat in patients who were refractory to magnesium oxide. Furthermore, it is considered that elobixibat succeeded in producing a therapeutic effect through its triple action in patients with CC who were refractory to intestinal secretagogues, which are single‐action laxatives. Elobixibat not only produced a significant improvement in SBM in patients whose SBM was unresponsive (<3 times/week) to treatment with magnesium or intestinal secretagogues, but also improved the sensation of incomplete bowel evacuation in patients with more frequent SBM (≥3 times/week). These results suggest that elobixibat may be useful for the treatment of constipation symptoms that are not improved with magnesium or intestinal secretagogues.

Moreover, the percentages of patients who discontinued stimulant laxatives and enemas/suppositories during the observation period were high. Elobixibat, as a replacement for stimulant laxatives or enemas, is expected to reduce the risk with these laxatives, such as acquired resistance and rectal mucosal injury,^39^, ^40^ while improving constipation symptoms.

Polypharmacy is a frequent problem with laxatives.^41^ In this study, improvements in constipation symptoms were observed even in patients who had previously used three or more types of laxatives, demonstrating the efficacy of elobixibat in patients who had been receiving multiple laxatives. The study also demonstrated the efficacy of elobixibat not only in those patients who had been receiving intestinal secretagogues and osmotic laxatives concomitantly but also those who had received intestinal secretagogues and stimulant laxatives concomitantly. It is presumed that the triple action of elobixibat produced therapeutic effects additional to those provided by these prior laxatives. Moreover, elobixibat was effective in 42 patients with CC who no longer required other laxatives after treatment with elobixibat began. Given that, overall, 43.9% of the prior laxatives were discontinued at the start of or after elobixibat treatment began, it will be important to examine whether elobixibat can help address polypharmacy, especially decrease in the number of laxatives used to treat constipation in elderly patients.

The adverse reactions that occurred in this study were like those in other clinical studies of elobixibat,^27^ with no adverse reactions raising new concerns or associated with any patient characteristics. Treatment discontinuations due to improvements in constipation symptoms occurred in 7.1% of the patients in this study. These discontinuations might have resulted from improvements in various constipation symptoms caused by physiological bowel movements, which were induced by bile acids increased by elobixibat.

The main limitation of this study was its single‐center retrospective design. The study was also unable to enroll more than half the patients receiving elobixibat owing to missing data on the frequency of bowel movements before and after elobixibat treatment. The results were obtained from a short duration of treatment, that is, 2 weeks. Most patients have developed a tolerance to laxatives after the initial use, making them less effective over time. Therefore, elobixibat may have demonstrated some superior efficacies in the first 2 weeks of treatment compared with the previously used laxatives, and it is unknown whether long‐term administration of elobixibat is more effective than other laxatives. In this study, patients with loose stools achieved moderate stool consistency, which contradicts elobixibat's water secretory action on the large intestinal lumen. This point would be difficult to explain in this study and may require further studies. We could not assess peristalsis, since it was difficult to obtain radiopaque markers and evaluate peristalsis based on other parameters collected in this study. There was no data of defecation desire in this study. Therefore, the effect of elobixibat on defecation desire was not evaluable. Additionally, we did not evaluate outcomes such as quality of life. In addition, the study was conducted only in Japan, and the results may differ for different races and backgrounds.

In conclusion, elobixibat was effective in terms of the frequency of bowel movements, stool form, and abdominal symptoms in patients who were refractory to other laxatives, irrespective of previous laxative therapy or patient characteristics. The study demonstrated the efficacy of elobixibat in the treatment of CC based on its triple action, it will be important to examine whether elobixibat can help address polypharmacy.

## Supporting information


**Table S1.** Cross‐tabulation of each constipation parameter before and after elobixibat treatment.
**Table S2.** Cross‐tabulation of the sensation of incomplete bowel evacuation (yes/no) by subgroup.

## Data Availability

The datasets generated during and/or analyzed during the current study are available from the corresponding author on reasonable request.
