# Supplementary material for: Efficacy and safety of elobixibat in combination with or switched from conventional treatments of chronic constipation: A retrospective observational study
Source: JGH Open. 2024 Aug 26;8(8):e70019. doi: 10.1002/jgh3.70019 (PMC11347617; doi:10.1002/jgh3.70019)
Supplement: Supplementary file 1 — Table S1. Cross‐tabulation of each constipation parameter before and after elobixibat treatment. Table S2. Cross‐tabulation of the sensation of incomplete bowel evacuation (yes/no) by subgroup. [file JGH3-8-e70019-s001.docx]

**Supporting information**

**Table S1** Cross-tabulation of each constipation parameter before and after elobixibat treatment

| Parameter | Baseline | Week 2 | | |  |
| --- | --- | --- | --- | --- | --- |
|  |  | Yes | No | Total | P value |
| Sensation of incomplete bowel evacuation | Yes | 18 ( 22.5 ) | 42 ( 52.5 ) | 60 ( 75.0 ) | < 0.0001* |
|  | No | 0 ( 0.0 ) | 20 ( 25.0 ) | 20 ( 25.0 ) |  |
|  | Total | 18 ( 22.5 ) | 62 ( 77.5 ) | 80 |  |
| Straining | Yes | 4 ( 6.6 ) | 18 ( 29.5 ) | 22 ( 36.1 ) | 0.0001* |
|  | No | 1 ( 1.6 ) | 38 ( 62.3 ) | 39 ( 63.9 ) |  |
|  | Total | 5 ( 8.2 ) | 56 ( 91.8 ) | 61 |  |
| Abdominal pain | Yes | 5 ( 8.9 ) | 12 ( 21.4 ) | 17 ( 30.4 ) | 0.0201* |
|  | No | 3 ( 5.4 ) | 36 ( 64.3 ) | 39 ( 69.6 ) |  |
|  | Total | 8 ( 14.3 ) | 48 ( 85.7 ) | 56 |  |
| Abdominal distention | Yes | 7 ( 10.6 ) | 26 ( 39.4 ) | 33 ( 50.0 ) | < 0.0001* |
|  | No | 2 ( 3.0 ) | 31 ( 47.0 ) | 33 ( 50.0 ) |  |
|  | Total | 9 ( 13.6 ) | 57 ( 86.4 ) | 66 |  |
| Difficulty defecating | Yes | 14 ( 20.3 ) | 27 ( 39.1 ) | 41 ( 59.4 ) | < 0.0001* |
|  | No | 0 ( 0.0 ) | 28 ( 40.6 ) | 28 ( 40.6 ) |  |
|  | Total | 14 ( 20.3 ) | 55 ( 79.7 ) | 69 |  |
| Nausea | Yes | 6 ( 11.3 ) | 3 ( 5.7 ) | 9 ( 17.0 ) | 0.3173 |
|  | No | 1 ( 1.9 ) | 43 ( 81.1 ) | 44 ( 83.0 ) |  |
|  | Total | 7 ( 13.2 ) | 46 ( 86.8 ) | 53 |  |

Data represent n (%). Marginal probability of each parameter before and after treatment was calculated using McNemar’s test. *p < 0.05

**Table S2** Cross-tabulation of the sensation of incomplete bowel evacuation (yes/no) by subgroup

| Subgroup | Threshold | Baseline |  | Week 2 |  | P value |
| --- | --- | --- | --- | --- | --- | --- |
|  |  |  | Yes | No | Total |  |
| Age | ≥ 65 years | Yes | 15 ( 23.8 ) | 36 ( 57.1 ) | 51 ( 81.0 ) | < 0.0001 |
|  |  | No | 0 ( 0.0 ) | 12 ( 19.0 ) | 12 ( 19.0 ) |  |
|  |  | Total | 15 ( 23.8 ) | 48 ( 76.2 ) | 63 |  |
|  | < 65 years | Yes | 3 ( 17.6 ) | 6 ( 35.3 ) | 9 ( 52.9 ) | 0.0143 |
|  |  | No | 0 ( 0.0 ) | 8 ( 47.1 ) | 8 ( 47.1 ) |  |
|  |  | Total | 3 ( 17.6 ) | 14 ( 82.4 ) | 17 |  |
| Sex | Male | Yes | 6 ( 18.8 ) | 19 ( 59.4 ) | 25 ( 78.1 ) | < 0.0001 |
|  |  | No | 0 ( 0.0 ) | 7 ( 21.9 ) | 7 ( 21.9 ) |  |
|  |  | Total | 6 ( 18.8 ) | 26 ( 81.3 ) | 32 |  |
|  | Female | Yes | 12 ( 25.0 ) | 23 ( 47.9 ) | 35 ( 72.9 ) | < 0.0001 |
|  |  | No | 0 ( 0.0 ) | 13 ( 27.1 ) | 13 ( 27.1 ) |  |
|  |  | Total | 12 ( 25.0 ) | 36 ( 75.0 ) | 48 |  |
| Weekly SBM before elobixibat treatment | ≥ 3 times/week | Yes | 9 ( 23.7 ) | 20 ( 52.6 ) | 29 ( 76.3 ) | < 0.0001 |
|  |  | No | 0 ( 0.0 ) | 9 ( 23.7 ) | 9 ( 23.7 ) |  |
|  |  | Total | 9 ( 23.7 ) | 29 ( 76.3 ) | 38 |  |
|  | < 3 times/week | Yes | 9 ( 21.4 ) | 22 ( 52.4 ) | 31 ( 73.8 ) | < 0.0001 |
|  |  | No | 0 ( 0.0 ) | 11 ( 26.2 ) | 11 ( 26.2 ) |  |
|  |  | Total | 9 ( 21.4 ) | 33 ( 78.6 ) | 42 |  |
| BSFS before elobixibat treatment | 1 - 2 | Yes | 7 ( 23.3 ) | 17 ( 56.7 ) | 24 ( 80.0 ) | < 0.0001 |
|  |  | No | 0 ( 0.0 ) | 6 ( 20.0 ) | 6 ( 20.0 ) |  |
|  |  | Total | 7 ( 23.3 ) | 23 ( 76.7 ) | 30 |  |
|  | 3 - 5 | Yes | 4 ( 17.4 ) | 11 ( 47.8 ) | 15 ( 65.2 ) | 0.0009 |
|  |  | No | 0 ( 0.0 ) | 8 ( 34.8 ) | 8 ( 34.8 ) |  |
|  |  | Total | 4 ( 17.4 ) | 19 ( 82.6 ) | 23 |  |
|  | 6 - 7 | Yes | 1 ( 14.3 ) | 2 ( 28.6 ) | 3 ( 42.9 ) | 0.1573 |
|  |  | No | 0 ( 0.0 ) | 4 ( 57.1 ) | 4 ( 57.1 ) |  |
|  |  | Total | 1 ( 14.3 ) | 6 ( 85.7 ) | 7 |  |
| Conditions: Cardiovascular diseases | Yes | Yes | 12 ( 32.4 ) | 18 ( 48.6 ) | 30 ( 81.1 ) | < 0.0001 |
|  |  | No | 0 ( 0.0 ) | 7 ( 18.9 ) | 7 ( 18.9 ) |  |
|  |  | Total | 12 ( 32.4 ) | 25 ( 67.6 ) | 37 |  |
| Conditions: Cancer | Yes | Yes | 2 ( 8.0 ) | 17 ( 68.0 ) | 19 ( 76.0 ) | < 0.0001 |
|  |  | No | 0 ( 0.0 ) | 6 ( 24.0 ) | 6 ( 24.0 ) |  |
|  |  | Total | 2 ( 8.0 ) | 23 ( 92.0 ) | 25 |  |
| Conditions: Diabetes mellitus | Yes | Yes | 5 ( 23.8 ) | 12 ( 57.1 ) | 17 ( 81.0 ) | 0.0005 |
|  |  | No | 0 ( 0.0 ) | 4 ( 19.0 ) | 4 ( 19.0 ) |  |
|  |  | Total | 5 ( 23.8 ) | 16 ( 76.2 ) | 21 |  |
| Conditions: Post-abdominal surgery | Yes | Yes | 4 ( 28.6 ) | 7 ( 50.0 ) | 11 ( 78.6 ) | 0.0082 |
|  |  | No | 0 ( 0.0 ) | 3 ( 21.4 ) | 3 ( 21.4 ) |  |
|  |  | Total | 4 ( 28.6 ) | 10 ( 71.4 ) | 14 |  |
| Conditions: Cerebral infarction  /hemorrhage | Yes | Yes | 4 ( 57.1 ) | 1 ( 14.3 ) | 5 ( 71.4 ) | 0.3173 |
|  |  | No | 0 ( 0.0 ) | 2 ( 28.6 ) | 2 ( 28.6 ) |  |
|  |  | Total | 4 ( 57.1 ) | 3 ( 42.9 ) | 7 |  |
| Conditions: Dementia | Yes | Yes | 3 ( 42.9 ) | 4 ( 57.1 ) | 7 ( 100.0 ) | 0.0455 |
|  |  | No | 0 ( 0.0 ) | 0 ( 0.0 ) | 0 ( 0.0 ) |  |
|  |  | Total | 3 ( 42.9 ) | 4 ( 57.1 ) | 7 |  |
| Conditions: Use of opioids | Yes | Yes | 0 ( 0.0 ) | 2 ( 66.7 ) | 2 ( 66.7 ) | 0.1573 |
|  |  | No | 0 ( 0.0 ) | 1 ( 33.3 ) | 1 ( 33.3 ) |  |
|  |  | Total | 0 ( 0.0 ) | 3 ( 100.0 ) | 3 |  |
| Number of types before the prescription of elobixibat | 1 type | Yes | 5 ( 26.3 ) | 10 ( 52.6 ) | 15 ( 78.9 ) | 0.0016 |
|  |  | No | 0 ( 0.0 ) | 4 ( 21.1 ) | 4 ( 21.1 ) |  |
|  |  | Total | 5 ( 26.3 ) | 14 ( 73.7 ) | 19 |  |
|  | 2 types | Yes | 4 ( 17.4 ) | 11 ( 47.8 ) | 15 ( 65.2 ) | 0.0009 |
|  |  | No | 0 ( 0.0 ) | 8 ( 34.8 ) | 8 ( 34.8 ) |  |
|  |  | Total | 4 ( 17.4 ) | 19 ( 82.6 ) | 23 |  |
|  | ≥ 3 types | Yes | 7 ( 21.9 ) | 18 ( 56.3 ) | 25 ( 78.1 ) | < 0.0001 |
|  |  | No | 0 ( 0.0 ) | 7 ( 21.9 ) | 7 ( 21.9 ) |  |
|  |  | Total | 7 ( 21.9 ) | 25 ( 78.1 ) | 32 |  |
| Osmotic laxatives initiated before the prescription of elobixibat | Yes | Yes | 9 ( 19.1 ) | 26 ( 55.3 ) | 35 ( 74.5 ) | < 0.0001 |
|  |  | No | 0 ( 0.0 ) | 12 ( 25.5 ) | 12 ( 25.5 ) |  |
|  |  | Total | 9 ( 19.1 ) | 38 ( 80.9 ) | 47 |  |
| Magnesium initiated before the prescription of elobixibat | Yes | Yes | 8 ( 20.0 ) | 22 ( 55.0 ) | 30 ( 75.0 ) | < 0.0001 |
|  |  | No | 0 ( 0.0 ) | 10 ( 25.0 ) | 10 ( 25.0 ) |  |
|  |  | Total | 8 ( 20.0 ) | 32 ( 80.0 ) | 40 |  |
| Stimulant laxatives initiated before the prescription of elobixibat | Yes | Yes | 10 ( 24.4 ) | 22 ( 53.7 ) | 32 ( 78.0 ) | < 0.0001 |
|  |  | No | 0 ( 0.0 ) | 9 ( 22.0 ) | 9 ( 22.0 ) |  |
|  |  | Total | 10 ( 24.4 ) | 31 ( 75.6 ) | 41 |  |
| Intestinal secretagogues initiated before the prescription of elobixibat | Yes | Yes | 7 ( 25.9 ) | 11 ( 40.7 ) | 18 ( 66.7 ) | 0.0009 |
|  |  | No | 0 ( 0.0 ) | 9 ( 33.3 ) | 9 ( 33.3 ) |  |
|  |  | Total | 7 ( 25.9 ) | 20 ( 74.1 ) | 27 |  |
| Chinese traditional medicines initiated before the prescription of elobixibat | Yes | Yes | 0 ( 0.0 ) | 6 ( 85.7 ) | 6 ( 85.7 ) | 0.0143 |
|  |  | No | 0 ( 0.0 ) | 1 ( 14.3 ) | 1 ( 14.3 ) |  |
|  |  | Total | 0 ( 0.0 ) | 7 ( 100.0 ) | 7 |  |
| Enemas or suppositories initiated before the prescription of elobixibat | Yes | Yes | 4 ( 22.2 ) | 14 ( 77.8 ) | 18 ( 100.0 ) | 0.0002 |
|  |  | No | 0 ( 0.0 ) | 0 ( 0.0 ) | 0 ( 0.0 ) |  |
|  |  | Total | 4 ( 22.2 ) | 14 ( 77.8 ) | 18 |  |
| Gastrointestinal prokinetic agents initiated before the prescription of elobixibat | Yes | Yes | 0 ( 0.0 ) | 2 ( 33.3 ) | 2 ( 33.3 ) | 0.1573 |
|  |  | No | 0 ( 0.0 ) | 4 ( 66.7 ) | 4 ( 66.7 ) |  |
|  |  | Total | 0 ( 0.0 ) | 6 ( 100.0 ) | 6 |  |
| Intestinal secretagogues and osmotic laxatives initiated before the prescription of elobixibat | Both used | Yes | 4 ( 28.6 ) | 6 ( 42.9 ) | 10 ( 71.4 ) | 0.0143 |
|  |  | No | 0 ( 0.0 ) | 4 ( 28.6 ) | 4 ( 28.6 ) |  |
|  |  | Total | 4 ( 28.6 ) | 10 ( 71.4 ) | 14 |  |
| Intestinal secretagogues and magnesium initiated before the prescription of elobixibat | Both used | Yes | 3 ( 23.1 ) | 6 ( 46.2 ) | 9 ( 69.2 ) | 0.0143 |
|  |  | No | 0 ( 0.0 ) | 4 ( 30.8 ) | 4 ( 30.8 ) |  |
|  |  | Total | 3 ( 23.1 ) | 10 ( 76.9 ) | 13 |  |
| Intestinal secretagogues and stimulant laxatives initiated before the prescription of elobixibat | Both used | Yes | 4 ( 25.0 ) | 7 ( 43.8 ) | 11 ( 68.8 ) | 0.0082 |
|  |  | No | 0 ( 0.0 ) | 5 ( 31.3 ) | 5 ( 31.3 ) |  |
|  |  | Total | 4 ( 25.0 ) | 12 ( 75.0 ) | 16 |  |
| Magnesium initiated before the prescription of elobixibat and weekly SBM before elobixibat treatment | Used and  ≥ 3 times/week | Yes | 4 ( 20.0 ) | 12 ( 60.0 ) | 16 ( 80.0 ) | 0.0005 |
|  |  | No | 0 ( 0.0 ) | 4 ( 20.0 ) | 4 ( 20.0 ) |  |
|  |  | Total | 4 ( 20.0 ) | 16 ( 80.0 ) | 20 |  |
|  | Used and  < 3 times/week | Yes | 4 ( 20.0 ) | 10 ( 50.0 ) | 14 ( 70.0 ) | 0.0016 |
|  |  | No | 0 ( 0.0 ) | 6 ( 30.0 ) | 6 ( 30.0 ) |  |
|  |  | Total | 4 ( 20.0 ) | 16 ( 80.0 ) | 20 |  |
| Intestinal secretagogues initiated before the prescription of elobixibat and weekly SBM before elobixibat treatment | Used and  ≥ 3 times/week | Yes | 3 ( 21.4 ) | 7 ( 50.0 ) | 10 ( 71.4 ) | 0.0082 |
|  |  | No | 0 ( 0.0 ) | 4 ( 28.6 ) | 4 ( 28.6 ) |  |
|  |  | Total | 3 ( 21.4 ) | 11 ( 78.6 ) | 14 |  |
|  | Used and  < 3 times/week | Yes | 4 ( 30.8 ) | 4 ( 30.8 ) | 8 ( 61.5 ) | 0.0455 |
|  |  | No | 0 ( 0.0 ) | 5 ( 38.5 ) | 5 ( 38.5 ) |  |
|  |  | Total | 4 ( 30.8 ) | 9 ( 69.2 ) | 13 |  |
| Prior laxatives, all of which were discontinued at the start of elobixibat treatment | Yes | Yes | 3 ( 18.8 ) | 8 ( 50.0 ) | 11 ( 68.8 ) | 0.0047 |
|  |  | No | 0 ( 0.0 ) | 5 ( 31.3 ) | 5 ( 31.3 ) |  |
|  |  | Total | 3 ( 18.8 ) | 13 ( 81.3 ) | 16 |  |

Data represent n (%). Marginal probability of each parameter before and after treatment was calculated using McNemar’s test.

SBM, spontaneous bowel movement; BSFS, Bristol stool form scale.
